# Immortalisation with hTERT Impacts on Sulphated Glycosaminoglycan Secretion and Immunophenotype in a Variable and Cell Specific Manner

**DOI:** 10.1371/journal.pone.0133745

**Published:** 2015-07-21

**Authors:** Tina P. Dale, Alice de Castro, Nicola J. Kuiper, E. Kenneth Parkinson, Nicholas R. Forsyth

**Affiliations:** 1 Institute for Science and Technology in Medicine, Keele University, Stoke-on-Trent, United Kingdom; 2 Research Centre for Clinical & Diagnostic Oral Sciences, Barts & The London School of Medicine & Dentistry, Queen Mary University of London, London, United Kingdom; University of Newcastle, UNITED KINGDOM

## Abstract

**Background:**

Limited options for the treatment of cartilage damage have driven the development of tissue engineered or cell therapy alternatives reliant on *ex vivo* cell expansion. The study of chondrogenesis in primary cells is difficult due to progressive cellular aging and senescence. Immortalisation via the reintroduction of the catalytic component of telomerase, *hTERT*, could allow repeated, longitudinal studies to be performed while bypassing senescent phenotypes.

**Methods:**

Three human cell types: bone marrow-derived stromal cells (BMA13), embryonic stem cell-derived (1C6) and chondrocytes (OK3) were transduced with *hTERT* (BMA13H, 1C6H and OK3H) and proliferation, surface marker expression and tri-lineage differentiation capacity determined. The sulphated glycosaminoglycan (sGAG) content of the monolayer and spent media was quantified in maintenance media (MM) and pro-chondrogenic media (PChM) and normalised to DNA.

**Results:**

*hTERT* expression was confirmed in transduced cells with proliferation enhancement in 1C6H and OK3H cells but not BMA13H. All cells were negative for leukocyte markers (CD19, CD34, CD45) and CD73 positive. CD14 was expressed at low levels on OK3 and OK3H and HLA-DR on BMA13 (84.8%). CD90 was high for BMA13 (84.9%) and OK3 (97.3%) and moderate for 1C6 (56.7%), expression was reduced in BMA13H (33.7%) and 1C6H (1.6%). CD105 levels varied (BMA13 87.7%, 1C6 8.2%, OK3 43.3%) and underwent reduction in OK3H (25.1%). 1C6 and BMA13 demonstrated osteogenic and adipogenic differentiation but mineralised matrix and lipid accumulation appeared reduced post *hTERT* transduction. Chondrogenic differentiation resulted in increased monolayer-associated sGAG in all primary cells and 1C6H (p<0.001), and BMA13H (p<0.05). In contrast OK3H demonstrated reduced monolayer-associated sGAG in PChM (p<0.001). Media-associated sGAG accounted for ≥55% (PChM-1C6) and ≥74% (MM-1C6H).

**Conclusion:**

In conclusion, *hTERT* transduction could, but did not always, prevent senescence and cell phenotype, including differentiation potential, was affected in a variable manner. As such, these cells are not a direct substitute for primary cells in cartilage regeneration research.

## Introduction

Cartilage damage due to injury or degenerative disease represents a significant challenge to the medical profession with limited treatment options available^,^[1]. Once compromised, this avascular, aneural tissue containing relatively small numbers of largely quiescent cells[2] usually fails to heal spontaneously, leading to long term tissue degradation[3]. This degradation is associated with poor function, joint pain and ultimately prosthetic joint replacement; this procedure is performed every 1.5 minutes in Europe, mainly due to osteoarthritis[1], with 15% of joint replacement surgeries being performed on those under 60 in the UK[4]. Although this surgery is frequently successful, the limited lifespan of prosthetic joints makes them a poor option for a younger demographic. Cell based therapies, which aim to promote intrinsic tissue regeneration, or to replace the degenerated tissue with engineered chondral or osteochondral constructs, are a promising alternative. To be successful these therapies need to recapitulate the proteoglycan/sGAG rich extracellular matrix (ECM) and restore tissue biomechanical properties. To date, therapies have often resulted in symptomatic improvements for patients[5] however they have not consistently resulted in hyaline tissue regeneration[6] which may impact on long term treatment efficacy.

Cell types currently under clinical investigation for cartilage repair include autologous chondrocytes and mesenchymal stem/stromal cells (MSCs). Initially evaluated in cartilage repair in 1994[7], autologous chondrocytes, with a mature native cartilage phenotype, are well suited. However they are available in limited quantities from a constrained donor site where tissue extraction may be associated with further donor site morbidity. They also require significant *ex-vivo* expansion which is associated with rapid dedifferentiation and a loss of chondrogenic phenotype[8]. Additionally there are as yet unanswered questions surrounding their clinical application at a time when, in older patients, many of the cells within the cartilage may be becoming senescent or apoptotic, particularly once the tissue is showing signs of osteoarthritis[9]. Chondrocyte senescence is increasingly implicated in the disease pathology with increased senescence associated β Galactosidase (SA βGal) activity in cells surrounding articular cartilage lesions, reduced mitotic activity and reduced telomere lengths, all correlating with increasing age[10].

As an alternative to chondrocytes, multipotent[11] mesenchymal stem/stromal cells, as described by Friedenstein *et al*[12], offer potentially greater proliferation, a more flexible response to differentiation cues and the prospect of an allogeneic therapy due to their immunomodulatory properties[13]. Unlike chondrocytes MSCs are also available from numerous tissues including several that would otherwise be discarded[14], removing the requirement for invasive surgery and healthy tissue removal. Both cell types have an adequate proliferative capacity for autologous cell therapies but show widespread variation in proliferation and then efficacy of differentiation potential between donors[15], particularly as the donor age increases[16]. Human embryonic stem cells (hESCs) have greater still proliferative and differentiation possibilities, due to their properties of self-renewal and pluripotency[17]. However numerous technical challenges concerning uniformity of differentiation and purity of cell populations must be resolved before these cells can be used safely due to the inherent capacity of undifferentiated cells to form teratomas. Despite these difficulties hESC derived cells are now entering early phase clinical trials[18].

A major hurdle to the use of any primary cell type is the progressive onset of replicative senescence. One cause of senescence is the shortening of telomeres, the TTAGGG nucleotide sequence repeats which cap linear mammalian chromosomes, preventing chromosomal degradation and fusion during mitotic DNA replication. Continued telomere erosion to a critically short length causes cells to become senescent as a result of DNA damage signalling events[19]. Additionally, more recently, telomeres have been proposed to be the repositories of irreparable DNA double strand breaks, especially in non-dividing cells owing to the reduced capacity of telomeres to engage non homologous end joining DNA repair[20][21]. Furthermore ectopic telomerase expression has been reported to slowly resolve the DNA lesions by an as yet unknown mechanism[22]. *In vivo*, endogenous telomerase expression is detectable only in stem and early progenitor cells, somatic cells in some rapidly renewing tissues and in cancerous cells, *in vitro* expression appears more restricted and is only consistently found in hESCs and cancer cells[23]. It has been demonstrated that replicative senescence can be avoided by the re-expression and activity of the telomerase reverse transcriptase catalytic subunit, *hTERT*[24–26] which can support the maintenance of telomere length whilst also preserving or even enhancing cell function[27].

The aim of this study was to compare the phenotype, particularly chondrogenic potential, of MSCs, chondrocytes and hESC derived MSC-like cells along with their corresponding *hTERT* transduced cell lines. *hTERT* was successfully introduced to all three cell types and prevented replicative senescence in chondrocytes and hESC derived MSC-like cells. Changes in cell phenotype were found in all three transduced cell lines including altered morphology, modifications in cell surface marker expression and alterations in differentiation capacity. Notably, transduced human chondrocytes lost chondrogenic capacity as a consequence of immortalisation.

## Materials and Methods

### Cell isolation and culture

Commercially sourced whole bone marrow aspirate (Lonza) was seeded at a density of 1x10^5^ mononuclear cells/cm^2^ in tissue culture flasks pre-coated with 10 ng/ml fibronectin (Sigma) in PBS. Cells were seeded in high glucose DMEM (4.5 g/L glucose) supplemented with 5% (v/v) foetal bovine serum (FBS), 1% (v/v) L-Glutamine (L-Glut), 1% (v/v) non-essential amino acids (NEAA) and 1% (v/v) Penicillin/Streptomycin/Amphotericin B (Lonza) and maintained in a 2% O_2_ atmosphere, to improve proliferation and chondrogenic potential[28]; a 50% media change was performed after one week and 100% media change at two weeks[29]. Following isolation MSC cells were designated BMA13. BMA13 and commercially sourced primary human articular (knee/hip) chondrocytes, OK3 (PromoCell), underwent routine media changes twice weekly with maintenance media (MM) consisting of DMEM supplemented with 10% (v/v) FBS, 1% (v/v) L-Glut, 1% (v/v) NEAA and 4 ng/mL bFGF (Peprotech) to enhance proliferation[30]. Embryonic stem cell clonally derived progenitor cells 1C6 were derived from the H1 embryonic stem cell line as described by Forsyth *et al*[31]. Cells were cultured in a MM of KO-DMEM (Life Technologies) supplemented with 10% (v/v) FBS, 1% (v/v) L-Glut, 1% (v/v) NEAA, 4 ng/mL bFGF and 100 nM dexamethasone (Sigma). A complete media change was performed twice weekly. All cells were sub-cultured enzymatically at confluence using 0.25% trypsin/EDTA (Lonza). Population doublings (PD) of cells were estimated based upon the split ratio of cells at sub-culture, cells were assumed to be senescent when the cumulative PD plateaued and cells no longer achieved 100% confluence after at least 30 days in culture.

### Retroviral transduction with *hTERT*

To increase cellular replicative potential, *hTERT* retroviral transduction was performed in all three cell types. BMA13 and OK3 cells were seeded at 5x10^4^ cells per well and 1C6 at 2.5x10^4^ per well in 6 well culture plates and cultured for 48 hours prior to infection. The Phoenix A packaging cell line was infected with either pBABE-hTERT or pBABE (empty vector) retroviral vectors[24]. Supernatant containing virus was filtered with a 0.45 μm syringe filter and used to spinfect target cells in the presence of 5 μg/ml polybrene by centrifuging the pre-gassed (10% CO_2_/90% air) and sealed plates for 1 hour at 10 g at 32°C. After 6 hours the infection process was repeated with fresh viral supernatant. Selection of successfully transduced cells was begun 24 hours after infection with medium supplemented with G418 (Sigma) at 1 mg/mL for two weeks with hTERT, empty vector and untransfected, plates.

### Gene expression analysis

Expression of *hTERT* was determined using reverse transcription polymerase chain reaction (RT-PCR). RNA was extracted from cells using the RNEasy minikit and QIAshredder homogenisation minispin columns (Qiagen) following the manufacturer’s protocol. RNA extracts were amplified using one step RT-PCR with the SuperScript III One-Step RT-PCR Platinum *Taq* HiFi kit (Life Technologies). RT-PCR was carried out with custom primers (Life Technologies) for the housekeeping gene *β-Actin*, and *hTERT* with primer sequences: *β-Actin*: forward: GCCACGGCTGCTTCCAGC, reverse: AGGGTGTAACGCAACTAAGTC, *hTERT*: forward: GCAGCTCCCATTTCATCAGC, reverse: AGGATGGTCTTGAAGTCTG. RT–PCR products were separated and viewed on 2% agarose gels with 0.5 μg/mL ethidium bromide (Sigma).

### Flow cytometry immunophenotyping

Cells were trypsinised, counted and re-suspended in flow cytometry buffer (R & D systems) for 15 minutes and approximately 1X10^5^ cells aliquoted into 1.5 mL microcentrifuge tubes, centrifuged at 300 g for 10 minutes and the supernatant discarded. Phycoerythrin conjugated antibodies (CD14, CD19, CD34, CD45, CD73, CD90 and CD105, HLA-DR, IgG_1_ and IgG2_a_ isotype controls (Miltenyi Biotech)) in flow cytometry buffer were used to re-suspend cell pellets followed by incubation in the dark at 4°C for 10 minutes. A 10X volume of buffer was added and cells centrifuged at 300 g for 10 minutes, supernatant discarded and the cells re-suspended in flow cytometry buffer. At least 50,000 events were acquired on a Beckton Dickinson FSC500 flow cytometer. Percentage positive events were determined using gates to exclude 99% of the appropriate isotype control events.

### Senescence associated β-Galactosidase staining

A senescent phenotype was confirmed by SA βGal staining[32] using an SA βGal histochemical staining kit (Sigma) according to the manufacturer’s protocol. Staining was performed for 16 hours at 37°C after which the staining mixture was removed and the cells washed with PBS. Cells were counterstained with haematoxylin prior to the acquisition of photomicrographs and cells in 4 field of view for each cell type were counted for SAβGal activity using Fiji image analysis software[33].

### Tri-lineage differentiation of cells

To determine differentiation potential cells were seeded at 5x10^4^/cm^2^ in standard proliferation media overnight to allow attachment after which media were changed to differentiation media. Cells were then cultured in differentiation media for 20 days with media changes twice weekly. Osteogenesis was induced in cells using media supplemented with 50 μM ascorbic acid, 10 mM β-glycerophosphate and 0.1 μM dexamethasone before being fixed with 95% methanol and staining with 1% alizarin red S for calcium deposits (Sigma). Adipogenesis was induced with 0.5 μM dexamethasone, 0.5 mM 3-isobutyl-1-methylxanthine, 10 μg/mL insulin and 0.1 mM indomethacin. Cells were fixed with 4% (w/v) paraformaldehyde in PBS, washed with 60% (v/v) isopropanol before being stained with oil red O for intracellular lipid droplets. Chondrogenic differentiation was induced using pro-chondrogenic media (PChM) which consisted of basal media with reduced FBS (1% (v/v) FBS) further supplemented with, 1% (v/v) ITS, 1% (v/v) sodium pyruvate, 0.1 μM dexamethasone, 50 μM ascorbic acid, 40 μg/mL L-proline (Sigma) and 10 ng/mL TGF-β_3_. Cells were fixed with 95% methanol and stained with alcian blue (pH 1.5) overnight for sulphated glycosaminoglycan accumulation.

### Sample preparation for DNA and total sGAG analyses

Chondrogenic differentiation was induced using PChM as described. Cells were seeded in 24 well tissue culture plates in MM and incubated overnight to allow attachment. MM were then substituted with fresh MM or PChM and changed regularly throughout the experiment, all media were collected for sGAG analysis.

At days 0 and 20 media was removed and cell monolayer digested with 250 μg of proteinase K in 200 μL of 100 mM ammonium acetate for 24 hours at 60°C. Samples were precipitated with ice cold ethanol overnight at -20°C, centrifuged at 13200 rpm to pellet the precipitate and the ethanol supernatant discarded. Pellets were allowed to dry then re-dissolved in 125 μL of 100 mM ammonium acetate for PicoGreen and DMMB analysis.

Collected media was treated with 250 μg of 2.5 mg/mL proteinase K per mL overnight followed by the addition of 1 mL of ice-cold ethanol and precipitation at -20°C overnight. Precipitate was pelleted by centrifugation at 17,000 g for 15 minutes and the ethanol supernatant discarded before the pellet was dried and re-dissolved in 125 μL of 100 mM ammonium acetate for DMMB analysis.

### Total DNA quantification by PicoGreen double stranded DNA assay

The Quant-iT PicoGreen double stranded DNA assay (Life Technologies) was carried out according to the manufacturer’s protocol. Duplicate sample aliquots were diluted 1 in 10 in TE buffer in 96 well microplates prior to incubation with PicoGreen working solution in the dark. Plates were read using a Synergy 2 plate reader (Biotek) (excitation 480 nm, emission 520 nm). Concentrations were determined using a lambda DNA calibration curve.

### Total sGAG quantification by DMMB assay

Samples dissolved in 100 mM ammonium acetate were used for DMMB total sGAG quantification[34] and were aliquoted in duplicate into a 96 well microplate followed by transfer into a Synergy 2 plate reader. An automated dispense unit was then used to dispense 200 μL of DMMB solution (16 mg DMMB, 0.76 g glycine, 0.595 g NaCl, 23.75 mL 0.1 M HCl, distilled H_2_O to 250 mL) to a single well followed by determination of the absorption at 530 nm. All wells were processed in this manner to maximise reproducibility and minimise the potential for precipitation of the DMMB-sGAG complex.

### Statistical analysis

Data are expressed as mean ± standard deviation. Statistical analysis of SAβGal staining was by one way ANOVA with Tukey’s multiple comparisons. Statistical analysis of sGAG production was by repeated measures two-way ANOVA with Bonferroni post-tests of chondrogenic supplemented samples compared to non-supplemented controls using Graphpad Prism 6.01, Graphpad, San Diego California, USA. Results were deemed to be statistically significant when *p* <0.05.

## Results

### The impact of *hTERT* transduction

**Fig 1:**
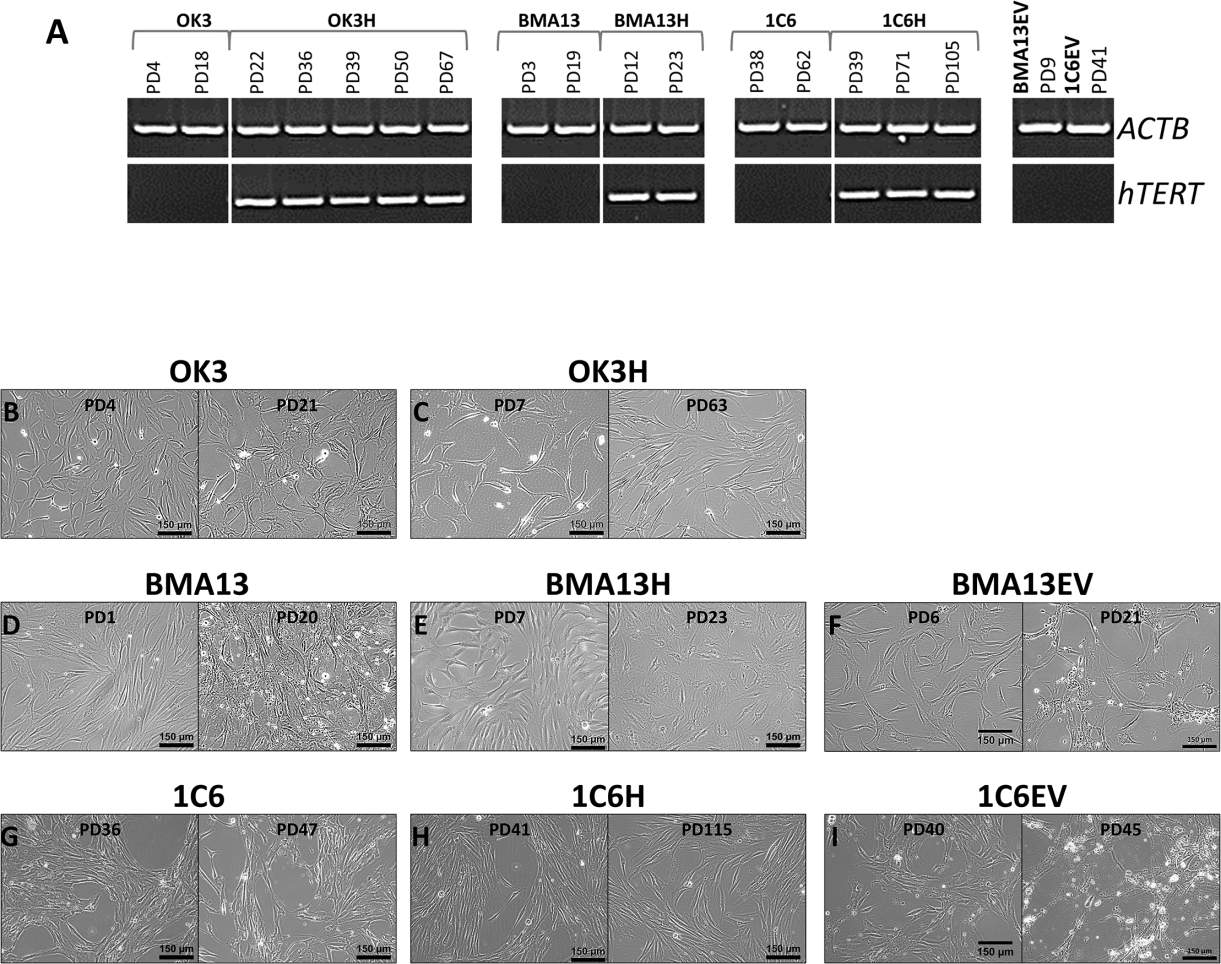
*hTERT* expression and impact on cell morphology. (A) Expression of the housekeeping gene *ACTB* and *hTERT* determined by RT-PCR in cells following recovery post transduction and with expansion in continuous cell culture. (B-I) Phase contrast micrographs of cells in monolayer culture pre- (B, D and F) and post- (C, E and G) transduction with *hTERT* or with empty vector (F and I) Scale = 150 μm, exact PD as indicated in image.

Following *hTERT* transduction RT-PCR confirmed *hTERT* expression in all three transduced cell types, whilst expression was absent in the non-transduced cell populations and the empty vector transduced cells BMA13EV and 1C6EV (Fig 1A). Expression in transduced cells was detected following initial expansion of transduced cells in culture post transduction, and was maintained in continuous culture.

Optical phase contrast microscopy was used to examine the cell morphology of parental and transduced cells. 1C6H (Fig 1C), 1C6EV (Fig 1I) and OK3H (Fig 1H) and BMA13EV (Fig 1F) retained similar morphologies post transduction to their respective non-transduced cells 1C6 (Fig 1B) and OK3 (Fig 1D). 1C6 and 1C6H both proliferated as closely aligned, colony-like clusters of small bipolar cells. OK3 and OK3H had a fibroblastic morphology consistent with that of dedifferentiated chondrocytes. However, changes were observed with BMA13H (Fig 1E) in comparison to BMA13 (Fig 1F). BMA13 had the expected spindle shaped MSC morphology eventually forming closely aligned colonies, however, BMA13H whilst initially resembling the parental cells later presented as a more heterogeneous population with cells frequently having a larger more flattened morphology with multiple cell processes.

**Fig 2:**
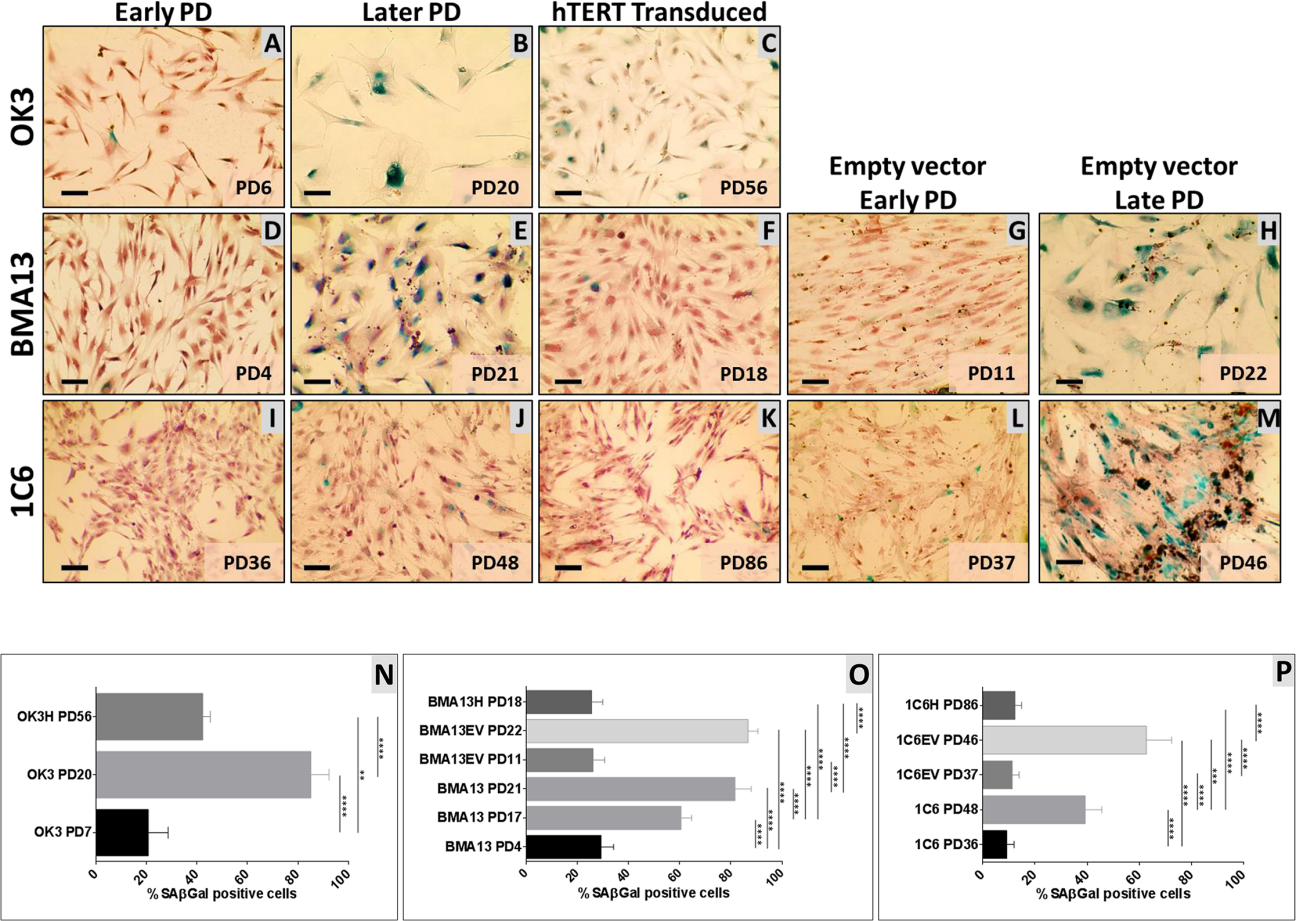
The effect of *hTERT* expression on senescence associated β Galactosidase (SA βGal) activity. (A-M) SA βGal activity in early and late PD non-transduced and empty vector transduced cells, and late PD *hTERT* transduced cells. Scale = 100 μm, exact PD as indicated in image. (N-P) Percentage of cells stained positively for SA βGal activity, mean n = 4 ± SD. (** p≤0.01, **** p≤0.0001)

*hTERT* transduction conferred gains in proliferative capacity in 1C6H, where cells proliferated for > 100 PD compared to 60 PD in 1C6 cells. OK3H achieved >60 PD compared to 20 PD with OK3 cells, although OK3H cells displayed a substantial period of reduced proliferation, at around 35 PD, followed by recovery to earlier rates. BMA13H proliferation ceased after only a slightly increased number of PD to PD31 compared to PD23 achieved with BMA13. Expansion with cryopreserved BMA13H (cryopreserved at PD8) resulted in a further reduced capacity for proliferation to levels matching BMA13 so further experiments were performed with BMA13H cells at PD16-20. 1C6H and OK3H have both continued to proliferate beyond PD107 and PD60 respectively (S1 Fig). Cells were stained for SA βGal activity (Fig 2A–2M) which was then quantified (Fig 2N–2P); all early PD non-transduced cells stained weakly for SA βGal, this was significantly (p≤0.0001) increased in late PD non-transduced and empty vector cells with these strongly stained cells often also having an altered morphology whereby cells were larger and rounder. Later PD *hTERT* transduced 1C6H and BMA13H had minimal staining that was comparable to early PD non-transduced cells. As BMA13H were stained at a slightly lower PD than BMA13 at PD21 an earlier culture of BMA13, at PD17, was also stained to ensure that senescence had not rapidly increased in BMA13 within the last few PD. Quantification of the cells at PD17 resulted in 60.5 ± 7.9% positive staining, significantly higher (p≤0.0001) than basal levels and PD 18 BMA13H. OK3H cultures displayed both SA βGAL positive and negative cells resulting in a population that stained significantly (p≤0.01) higher than parental low PD cells but remained significantly lower than late PD parental cells (p≤0.0001).

### Cell immunophenotype

**Fig 3:**
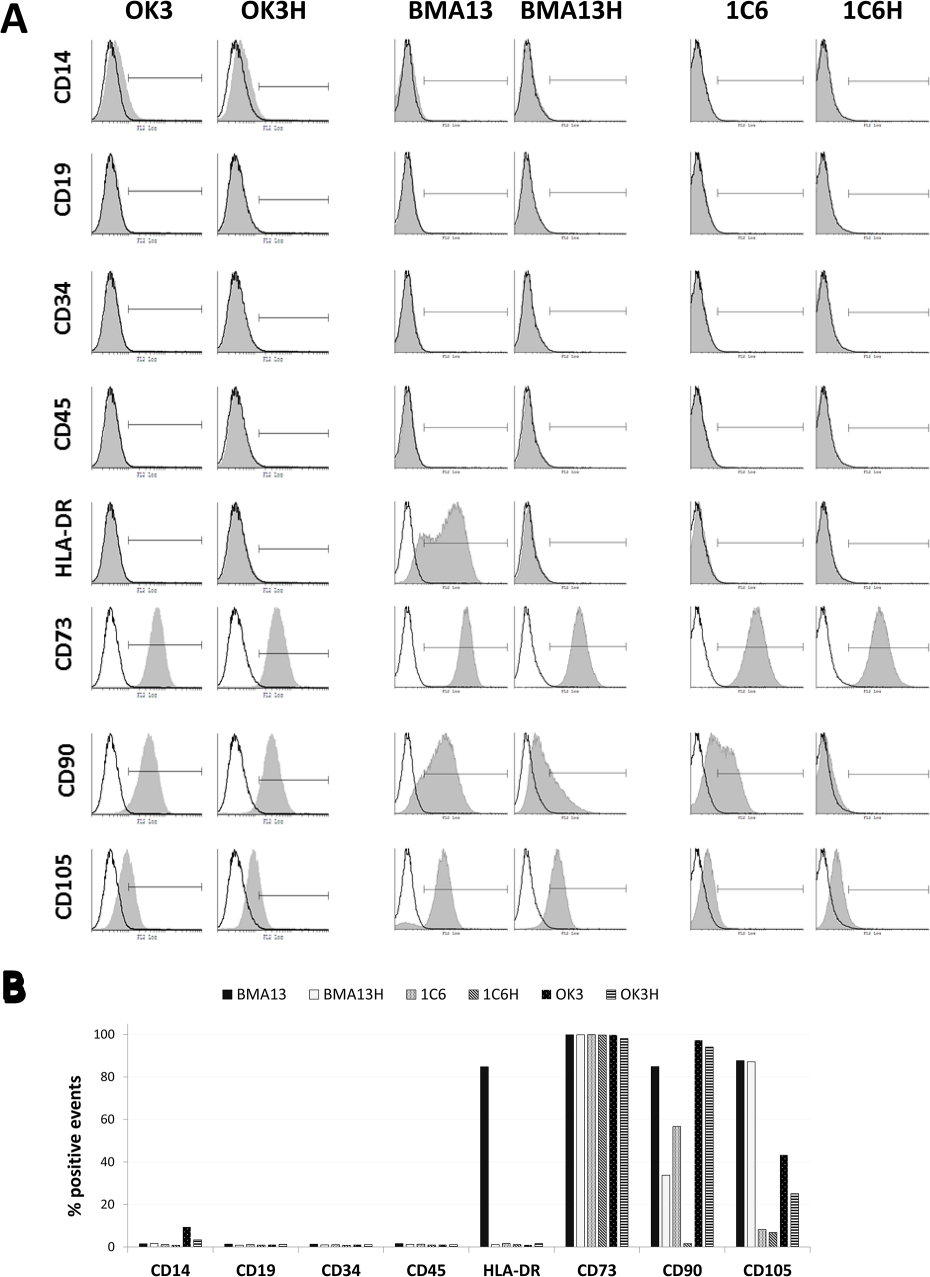
Flow cytometry analysis of cell surface marker expression. (A) Expression of surface markers CD14, CD19, CD34, CD45, HLA-DR, CD73, CD90, CD105 (filled) with relevant isotype control (unfilled). (B) Quantification of the percentage of positive events compared to the relevant isotype control. Markers set to exclude 99% of isotype control events; ≥ 4x10^4^events were collected per sample. OK3 was tested at PD10, OK3H at PD50, BMA13 at PD6, BMA13H at PD18, 1C6 at PD44 and 1C6H at PD118.

All cell types were analysed by flow cytometry for expression of haematopoietic (CD14, CD19, CD34, CD45 and HLA-DR) and mesodermal (CD73, CD90, CD105) cell surface markers (Fig 3).

All cells were negative (<2% positive compared to the isotype control) for leukocyte markers CD14, CD19, CD45 and the haematopoietic cell marker CD34 with the exception of slight positivity for CD14 in OK3 (9.4%) and OK3H (3.4%). HLA-DR expression was also absent in all but BMA13 cells where 84.8% of events were positive. The BMA13 cells were cultured to passage 4 in the presence of bFGF and similar HLA-DR positive staining has been noted in these conditions by Sotiropoulou *et al*[35]. CD73 expression was high in all cell types (>98%) however variation in immunophenotype was noted with CD90 and CD105 according to both cell type and immortalisation status. BMA13 expression of CD90 and CD105 was 84.9% and 87.7%, respectively, whereas transduced BMA13H maintained CD105 expression (87.1%) but displayed reduced CD90 expression (33.7%). 1C6 cells expressed moderate CD90 (56.7%), which was reduced post-immortalisation (1.6%), and low CD105 (8.2% vs. 6.8% (1C6H). OK3 and OK3H displayed high expression of CD73 and CD90 (>94%) and moderate expression of CD105 (43.1%) which was reduced inOK3H (25.1%).

### Cell differentiation

**Fig 4:**
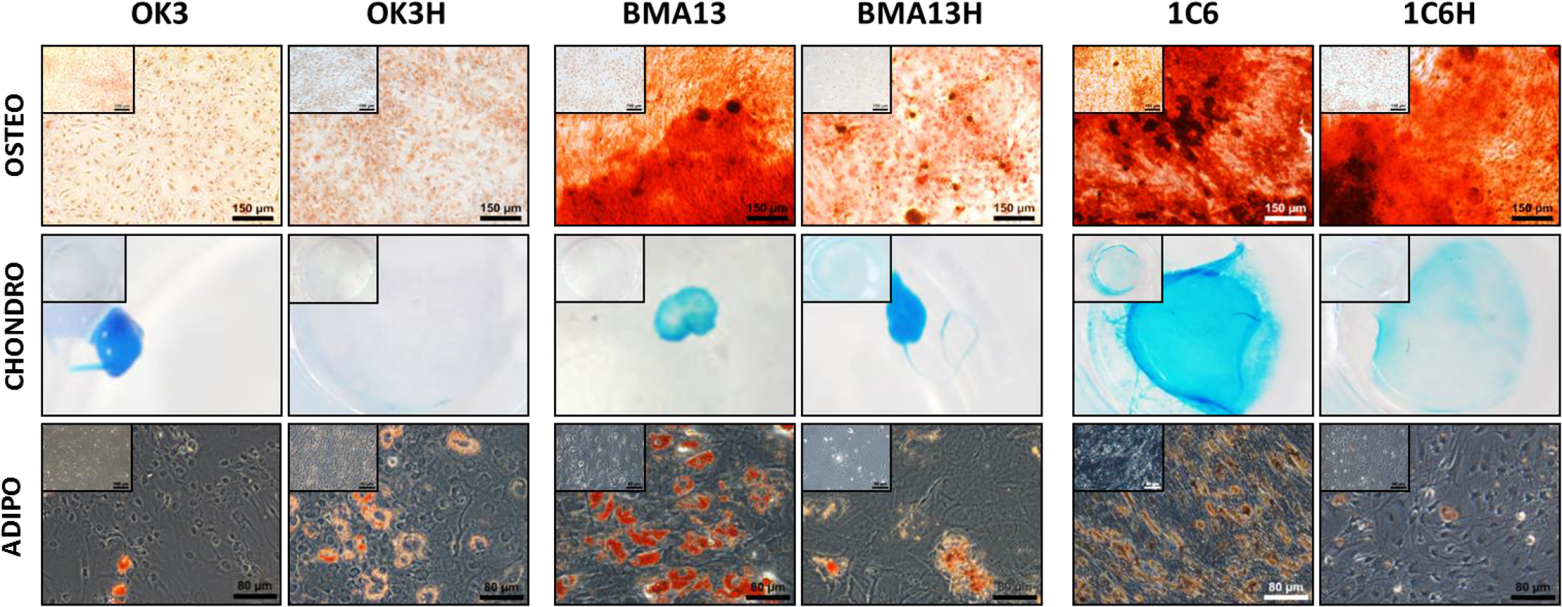
Tri-lineage differentiation capacity of cells over 21 days. Cells were cultured in monolayer for 21 days in the presence of the appropriate pro-differentiation supplements followed by fixation and staining. Osteogenic samples were stained with alizarin red for calcium deposits, scale = 150 μm. Chondrogenic samples were stained with alcian blue for SGAGs, images are from 1 well of a 24 well plate. Chondrogenic medium frequently caused cells to partially or fully separate from the substrate and contract into pellet-like structures. Adipogenic samples are stained with oil red O for lipid droplets, scale = 80 μm. Images of controls cells cultured in standard maintenance media and stained as appropriate are inset for comparison.

Soluble media supplements were used to drive differentiation in cells followed by fixation, histological staining and imaging (Fig 4).

Under the influence of differentiation supplements 1C6 and BMA13 cells underwent osteogenesis, with positive alizarin red staining of mineralised areas; chondrogenesis, indicated by alcian blue sGAG staining; and adipogenesis, with oil red O stained lipid vesicles. OK3 cells did not produce mineralised matrix, stained strongly for sGAG and showed some accumulation of lipids. 1C6H and BMA13H also differentiated however staining of mineralised matrix and lipids appeared reduced. As with OK3, OK3H did not mineralise under osteogenic conditions, however the response to pro-chondrogenic supplements was greatly reduced compared to OK3 with little sGAG staining, along with an apparent increase in lipid accumulation.

### Quantification of sGAG production during *in vitro* chondrogenesis

**Fig 5:**
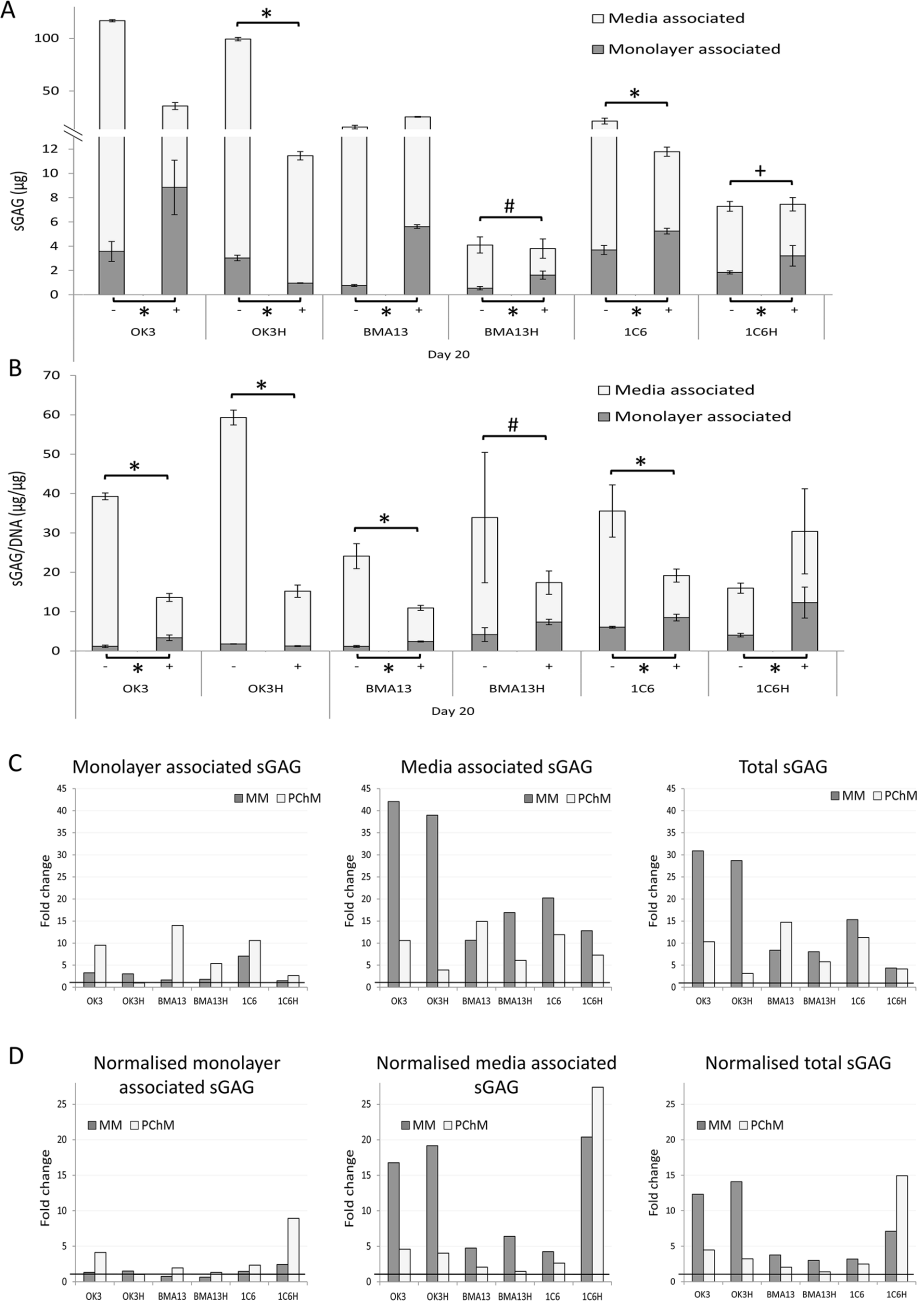
Quantification of sulphated glycosaminoglycan (sGAG) production and retention. (A) DMMB quantification of sGAG in both the media fraction and monolayer fraction after culture in either MM (-) or PChM (+) for 20 days. (B) Normalisation of sGAG to DNA at Day 20 in MM (-) or PChM (+). (C) Fold change in sGAG at Day 20 compared to levels determined at Day 0 in monolayer (left), media (middle) and in total (right). (D) Fold change in DNA normalised sGAG at Day 20 compared to Day 0 in monolayer (left), media (middle) and in total (right). Bold line at a fold change of 1 indicates no change compared to Day 0 levels. # p<0.05, + p<0.01, *p<0.001 (mean n = 3 ±SD). Experiments were performed at OK3 PD6, OK3H PD50, BMA13 PD6, BMA13H PD16, 1C6 PD 42 and 1C6H PD84.

sGAG associated with both monolayer and culture media was compared in *hTERT* transduced and parental cell types following 20 days of culture in the presence of MM or PChM. All cultures displayed an increase in total sGAG after 20 days in culture compared to day 0 values irrespective of culture media conditions (Fig 5A and 5C). Larger total sGAG increases were detected when cells were cultured in MM than PChM in all cases excepting BMA13, with increases in sGAG in MM ranging from 4.4 fold over day 0 values in 1C6H to 30.9 fold in OK3. This compared to values in PChM ranging from 3.1 fold in OK3H to 14.7 fold in BMA13. All three non-transduced cell types had greater fold increases and total sGAG production than their corresponding *hTERT* transduced cell types.

By day 20 PChM resulted in greater amounts of monolayer associated sGAG (p<0.001) in all cases except OK3H where monolayer sGAG was higher in MM (p<0.001). Concomitantly the media associated fraction was significantly reduced in PChM in all but BMA13. The proportion of total sGAG associated with the monolayer by day 20 ranged from 3.0% (OK3H) to 25.2% (1C6H) in MM and 8.4% (OK3H) to 44.5% (1C6) in PChM.

The BMA13EV response was comparable to the primary cell response (S2 Fig) with a significant (p≤0.001) increase in monolayer associated sGAG concurrent with a significantly decreased media associated sGAG, resulting in an overall reduction in total sGAG in PChM. In contrast 1C6EV had a much lower response although this still followed the same trends with only a 1.4 fold increase in monolayer associated sGAG with the difference between the sGAG monolayer content in the two media types not reaching significance.

As with absolute levels of sGAG, higher normalised monolayer associated sGAG (p<0.001) was found in all cells except OK3H, concurrent with a decreased media associated fraction, in PChM compared to MM (Fig 5B and 5D). Normalisation of sGAG to DNA (μg/μg) enables changes in sGAG due to altered cell numbers as a result of cell proliferation or cell death to be separated from changes in sGAG synthetic activity. 1C6 and BMA13 had large fold increases in absolute monolayer associated sGAG but low normalised sGAG indicative of a high degree of cell proliferation associated with the increased sGAG. In contrast high normalised sGAG compared to relatively low absolute levels were seen in 1C6H in PChM. This was as a result of a lower DNA content (below day 0 levels), and not solely as a result of high sGAG synthesis, leading to a correspondingly large fold change in normalised monolayer associated sGAG.

## Discussion

This study aimed to quantify the sGAG produced, a key measure of chondrogenic response, in a simple pro-chondrogenic *in vitro* environment using three alternative cell sources: MSCs, chondrocytes and ESC derived cells, all with potential for cartilage tissue engineering applications. The ECM glycosaminoglycan content represents a responsive marker for chondrogenesis due to its relatively rapid *in vivo* turnover rate compared to other cartilage matrix components[36]. We have previously subjected the parental cell types OK3, 1C6, and hMSC to RT-PCR analysis for several genes, including the COL2A1, transcript and found a poor correlation between chondrogenic induction, gene expression and ECM protein content [37], as have others[38].

We investigated the immortalisation of these cells with the catalytic subunit of telomerase[39–41] to improve their longevity and usefulness as a research tool. The expression of the catalytic subunit, *hTERT*, rather than the other components of telomerase (*hTR* and *TP1*) has been determined to be the limiting step in telomerase activity with strong correlation between *hTERT* expression and telomerase activity[42]. This immortalization approach has particular applicability for cartilage based cell therapies as in addition to the phenomenon of replicative senescence, primary chondrocytes undergo rapid de-differentiation during *in vitro* culture with a corresponding loss of phenotype[8][43] including reduced expression of chondrocyte associated genes[44] and reduced responsiveness to pro chondrogenic signals[45], making any long term study of these cells challenging.

Low levels of telomerase activity have occasionally been detected in human chondrocyte and MSC cell cultures[46][47]. Our results were in accordance with the majority of previous literature and telomerase expression was absent in the primary non-transduced MSCs BMA13[48–52], chondrocytes OK3[53], and embryonic derived 1C6[31]. *hTERT* transduction resulted in lasting *hTERT* gene expression in all three cell types. However, despite this, we noted a variable response with respect to proliferative potential where 1C6H was the only cell type to undergo uninterrupted proliferation. The minimal changes in proliferative capacity seen in BMA13H are in contrast to most other publications reporting successful immortalisation of MSCs[54–56] although some report similar results to ours with a small gain in proliferation capacity but not immortalisation[57]. The previous track record for immortalisation success indicates that the cessation of growth in our cells may be due to a mechanism other than telomere shortening. A strong candidate in our case would be oxidative stress; the cells were conditioned to a 2% oxygen environment having been recovered and expanded in 2% however the transduction and selection procedures were performed at a standard ambient oxygen level (21%) exposing the cells to a significant period of hyperoxia. Oxidative stress during exposure to higher ambient O_2_ levels is a known risk factor for stress induced cellular senescence[58,59]. The 1C6 cells are known to be particularly sensitive to increased oxygen levels[31]; 1C6EV was observed to senescence prematurely in comparison to 1C6H despite both having been exposed to the same hyperoxic environment during transduction. However the presence of TERT has been demonstrated to improve cellular resistance to stressors via a non-canonical mechanism that is independent to telomere lengthening[60].

There are a limited number of reports describing chondrocyte immortalisation, particularly in human cells. Earlier experiments used viral proto-oncogenes including the simian virus 40 large T antigen (SV40-Tag)[61] to immortalise cells and described varying impact on cell phenotype and a complex inverse relationship between cell proliferation and ECM synthesis, whereby only slowly proliferating cells exhibited chondrogenic ECM synthesis[62][63]. Immortalisation of chondrocytes with *hTERT* has been described, with assessment of semi-quantitative changes in aggrecan and collagen II gene expression. However, ECM formation via the production and secretion of proteoglycan or proteins was not determined. Furthermore, although the cells appeared to retain some chondrogenic capacity aggrecan and collagen II expression were reduced compared to control chondrocytes[53]. However, the immortalisation process in chondrocytes may be more complex as others have determined a requirement for secondary transduction with human papillomavirus 16 oncogenes E6 and E7[64]. In low oxygen culture conditions similar to those employed here only one of three lines gained proliferative potential following *hTERT* transduction despite evidence of extended telomeres in all three[65]. These results along with the extended pause or “crisis period” experienced by OK3H suggest that a secondary event allowing bypass of p53 or p16^ink4a^ responses may have occurred during OK3H culture to allow continued proliferation, although further experiments will be required to determine this.

SA βGAL, a reported marker for senescent cells[32], in our samples resulted in the expected higher level of staining of increasing numbers of cells in cultures approaching their maximum number of population doublings. However, we observed that there was a low background level of activity in both earlier PD OK3 and BMA13, and more notably, high levels in OK3H although this was in conjunction with a population of cells with no detectable SA βGAL activity. It is possible that the OK3H is a mixed population with some cells undergoing senescence but alternatively it is well known that there are multiple culture conditions where SA βGAL, a lysosomal β-galactosidase[66], is found in proliferating cell populations including cells proliferating under stress and in regions of cell confluence[67][68]. The presence of SA βGAL activity despite ongoing proliferation is further evidence that mechanisms other than telomere length-dependent replicative senescence are active in our cultures. Similar results were found by Zhu *et al* [69] transfection with both *hTERT* and *CDK4* and further optimisation of culture conditions was necessary to produced differentiation competent immortalised muscle satellite cells[69].

There are no unique markers for the classification of cells as MSCs and, of necessity, MSCs are defined by a collection of cell surface markers and physical features. Specifically in 2006 a set of minimal criteria guidelines for the definition of cells as MSCs was published by Dominici *et al*[70] and this has since been used in many studies. This statement of the position of The International Society for Cellular Therapy specified that MSCs should be plastic adherent; express CD73, CD90 and CD105 and concomitantly lack expression of CD14 or CD11b, CD19 or CD79, CD34, CD45 and HLA-DR; and have tri-lineage mesodermal differentiation capacity[70]. The BMA13 MSCs that were used in this study expressed CD73, CD90 and CD105 and lacked expression of CD14, CD19, CD34 and CD45; however, moderate expression of HLA-DR was found. Up-regulation of HLA-DR expression has been observed previously on MSCs, more so when cells were isolated from whole bone marrow aspirate rather than separated mononuclear cells, and particularly in response to exposure to bFGF[71][72]_._ Interestingly we found that OK3 chondrocytes expressed CD73, CD90 and CD105, expression of MSC markers on chondrocytes has been reported previously[8][73] with a greatly increased expression of CD90 in response to monolayer culture[8]. Alternatively it has been proposed that these cells may be representative of a chondroprogenitor or MSC like cell type present within cartilage[73]. In addition to variable proliferation responses we also noted changes in immunophenotype linked to *hTERT*. CD90 expression was reduced in both BMA13H and 1C6H and CD105 was reduced in OK3H when compared to parental cells. CD73, CD90 and CD105 expression decreases with increased passage number in MSCs[74] so these changes may be indicative of a loss of MSC phenotype in BMA13H. A reduction in CD90 with increasing proliferation following transduction with CD90 has also been noted in human adipose derived stem cells[75].

During *in vitro* culture cells synthesise and secrete ECM molecules including sGAG containing proteoglycans to fill intercellular spaces[76]; our investigation found that all cell types, in pro-chondrogenic conditions induced by TGF-β3[77] or not, produced sGAG in measurable quantities. Interestingly total sGAG was generally determined to be higher in cells exposed to MM than in PChM, where much of the sGAG was found in the media rather than in association with the monolayer. Reports of sGAG quantities in media during *in vitro* culture vary ranging from negligible amounts[78] to a large fraction (35–90% depending on culture condition) of the total sGAG produced[79]. Media sGAG has been used as a proxy for total sGAG production[80] however our results do not support this, as we found large cell type dependent differences in the proportion of retained versus secreted sGAG. Evidence provided by others indicates that the losses to the media from the monolayer are probably as a result of the inability of the ECM to retain the proteoglycan and sGAG which then diffuse into the media[79], however proteoglycan degradation due to catabolic activity cannot be ruled out. Transduction with *hTERT* had a particularly detrimental effect on monolayer associated sGAG with OK3H in both absolute and normalised terms. This may be reflective of the particular difficulties that seem to be associated with chondrocyte transduction[64][65]. The transduction procedure is lengthy and cells continue to proliferate, and therefore dedifferentiate, during this process and may have passed a critical point for re-differentiation. The cells are also polyclonal, polyclonal expansion favours selection of rapidly growing cells over those with more favourable differentiation properties[81]; clonal expansion and colony characterisation and selection may enable the selection of a subpopulation of cells with a greater proliferative capacity.

To enable rapid assessment of the multiple cell types described herein experiments were performed in monolayer culture however it is accepted that a three dimensional environment can provide a more pro-chondrogenic influence[82][83]. As such further investigations are underway to determine whether a pellet culture system can restore greater chondrogenic capacity to our *hTERT* transduced cells.

## Conclusion

To regenerate articular cartilage, transplanted cells must produce large quantities of sGAGs that are retained within the area of damaged tissue. All three primary cell types assessed herein produced and retained significant amounts of sGAG in association with the monolayer in response to a pro-chondrogenic influence. Two (1C6H and BMA13H) of three *hTERT* transduced cell types exhibited similar responses to non-transduced cell types when sGAG was normalised to DNA level so may well have *in vitro* application as chondrogenic cell lines where large numbers of cells are required. However in contrast, the *hTERT* transduced chondrocyte line OK3H performed poorly in response to PChM and had no increase in sGAG retained in the monolayer. In all three *hTERT* lines cell immunophenotype differed from parental cell types. These findings indicate that whilst *hTERT* transduction can be useful in prolonging cell proliferation, its application may be associated with complex changes in cell phenotype and a loss of differentiated cell function can be induced in a variable and cell specific manner. In future, with further interventions to circumvent the problems we have identified herein, telomerised cells may still be a viable alternative.

## Supporting Information

S1 Fig Cumulative population doublings for primary and transduced cells. Cumulative population doublings for (A), OK3/OK3H (B) BMA13/BMA13H/BMA13EV and (C) 1C6/1C6H/1C6EV.
(TIF)

S2 Fig Quantification of sulphated glycosaminoglycan (sGAG) production and retention. (A) DMMB quantification of sGAG in both the media fraction and monolayer fraction after culture in either MM (-) or PChM (+) for 20 days. (B) Normalisation of sGAG to DNA at Day 20 in MM (-) or PChM (+). (C) Fold change in sGAG at Day 20 compared to levels determined at Day 0 in monolayer (left) and media (right). (D) Fold change in DNA normalised sGAG at Day 20 compared to Day 0 in monolayer (left) and media (right). Bold line at a fold change of 1 indicates no change compared to Day 0 levels. *p<0.001 (mean n = 3 ±SD). Experiments were performed at PD11 for BMA13EV and PD44 for 1C6EV. 1C6EV media fold change could not be calculated as D0 was below the limit of detection for the DMMB assay.
(TIF)
