# Supplementary figures and images for: Immortalisation with hTERT Impacts on Sulphated Glycosaminoglycan Secretion and Immunophenotype in a Variable and Cell Specific Manner
Source: PLoS One. 2015 Jul 21;10(7):e0133745. doi: 10.1371/journal.pone.0133745 (PMC4510558; doi:10.1371/journal.pone.0133745)

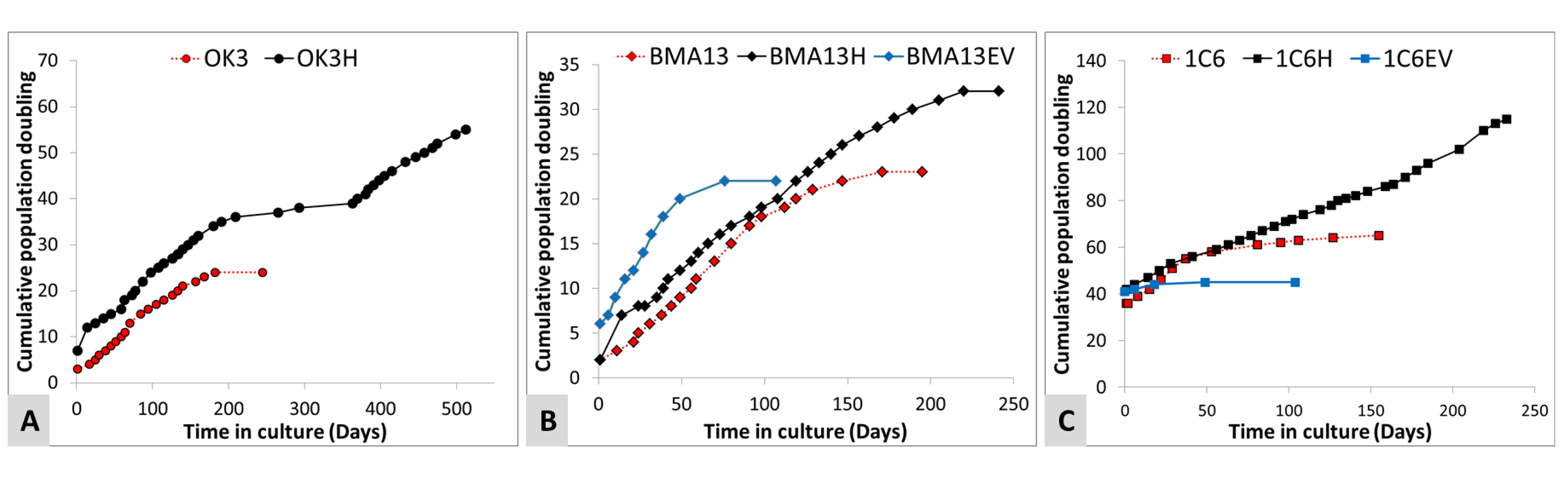

Supplement: S1 Fig — Cumulative population doublings for (A), OK3/OK3H (B) BMA13/BMA13H/BMA13EV and (C) 1C6/1C6H/1C6EV. (TIF) [file pone.0133745.s001.tif]

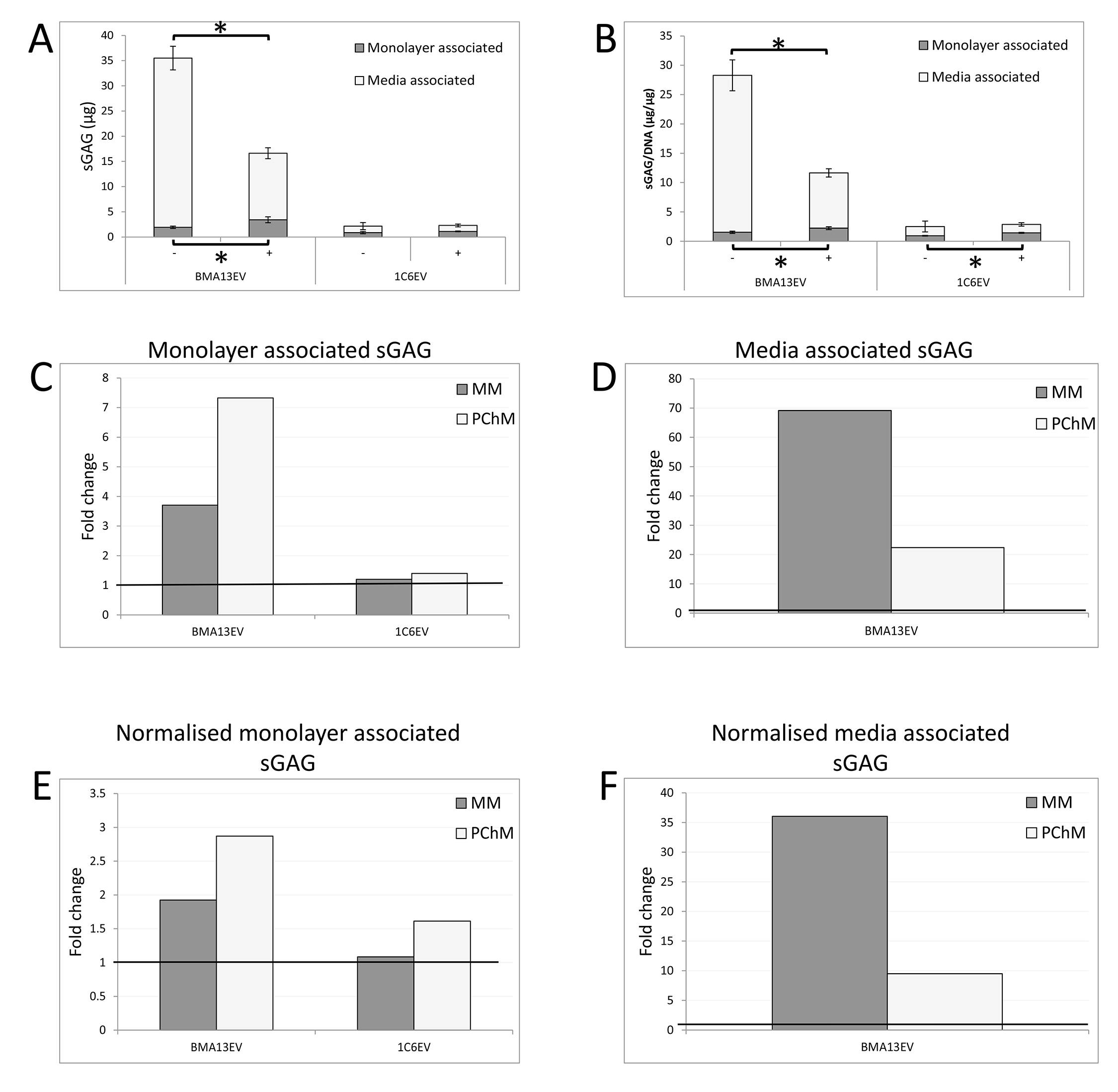

Supplement: S2 Fig — (A) DMMB quantification of sGAG in both the media fraction and monolayer fraction after culture in either MM (-) or PChM (+) for 20 days. (B) Normalisation of sGAG to DNA at Day 20 in MM (-) or PChM (+). (C) Fold change in sGAG at Day 20 compared to levels determined at Day 0 in monolayer (left) and media (right). (D) Fold change in DNA normalised sGAG at Day 20 compared to Day 0 in monolayer (left) and media (right). Bold line at a fold change of 1 indicates no change compared to Day 0 levels. *p<0.001 (mean n = 3 ±SD). Experiments were performed at PD11 for BMA13EV and PD44 for 1C6EV. 1C6EV media fold change could not be calculated as D0 was below the limit of detection for the DMMB assay. (TIF) [file pone.0133745.s002.tif]
